# Tetrahydrobiopterin deficiencies: Lesson from clinical experience

**DOI:** 10.1002/jmd2.12199

**Published:** 2021-02-01

**Authors:** Ayse Ergul Bozaci, Esra Er, Havva Yazici, Ebru Canda, Sema Kalkan Uçar, Merve Güvenc Saka, Cenk Eraslan, Hüseyin Onay, Sara Habif, Beat Thöny, Mahmut Coker

**Affiliations:** ^1^ Department of Pediatrics, Division of Pediatric Metabolism Ege University Faculty of Medicine Izmir Turkey; ^2^ Tepecik Research and Training Hospital, Department of Genetics Izmir Turkey; ^3^ Department of Radiology Ege University Faculty of Medicine Izmir Turkey; ^4^ Department of Medical Genetics Ege University Faculty of Medicine Izmir Turkey; ^5^ Department of Medical Biochemistry Ege University Faculty of Medicine Izmir Turkey; ^6^ Division of Metabolism University Children's Hospital Zurich and Children's Research Center Zurich Switzerland

**Keywords:** BH4, DHPR deficiency, hyperphenylalaninemia, l‐Dopa, PKU, tetrahydrobiopterin deficiencies

## Abstract

**Objectives:**

The present study describes clinical, biochemical, molecular genetic data, current treatment strategies and follow‐up in nine patients with tetrahydrobiopterin (BH4) deficiency due to various inherited genetic defects.

**Methods:**

We analyzed clinical, biochemical, and molecular data of nine patients with suspected BH4 deficiency. All patients were diagnosed at Ege University Faculty of Medicine in Izmir, Turkey and comprised data collected from 2006 to 2019. The diagnostic laboratory examinations included blood phenylalanine and urinary or plasma pterins, dihydropteridine reductase (DHPR) enzyme activity measurement in dried blood spots, folic acid and monoamine neurotransmitter metabolites in cerebrospinal fluid, as well as DNA sequencing.

**Results:**

Among the nine patients, we identified one with autosomal recessive GTP cyclohydrolase I (ar GTPCH) deficiency, two with 6‐pyruvoyl‐tetrahydropterin synthase (PTPS) deficiency, three with sepiapterin reductase (SR) deficiency, and three with DHPR deficiency. Similar to previous observations, the most common clinical symptoms are developmental delay, intellectual disability, and movement disorders. All patients received treatment with l‐dopa and 5‐hydroxytryptophan, while only the ar GTPCH, the PTPS, and one DHPR deficient patients were supplemented in addition with BH4. The recommended dose range varies among patients and depends on the type of disease. The consequences of BH4 deficiencies are quite variable; however, early diagnosis and treatment will improve outcomes.

**Conclusions:**

As BH4 deficiencies are rare group of treatable neurometabolic disorders, it is essential to diagnose the underlying (genetic) defect in newborns with hyperphenylalaninemia. Irreversible brain damage and progressive neurological deterioration may occur in untreated or late diagnosed patients. Prognosis could be satisfying in the cases with early diagnose and treatment.

**Synopsis**

It is essential to evaluate both newborns presenting with hyperphenylalaninemia and patients with unknown neurological origin in terms of BH4 deficiencies.

## 1 BACKGROUND

Tetrahydrobiopterin (BH4) deficiencies are rare group of neurometabolic disorders due to deficiency of enzymes involved in the biosynthesis and regeneration of BH4.^1^

BH4 is the essential cofactor for phenylalanine hydroxylase (PAH), tyrosine 3‐hydroxylase (TyrH), tryptophan‐5‐hydroxylase (TrpH), three isoforms of nitric oxide synthase (NOS), and glyceryl‐ether monooxygenase.^2^, ^3^ De novo biosynthesis and pterin salvage pathways are involved in BH4 biosynthesis. GTP cyclohydrolase I (GTPCH), 6‐pyruvoyl‐tetrahydropterin synthase (PTPS), and sepiapterin reductase (SR) enzymes play role in de novo biosynthesis pathway. In pterin salvage pathway, SR catalyses sepiapterin to the biologically inactive 7,8‐dihydrobiopterin (BH2) form with a two‐step reaction, which is converted to BH4 with dihydrofalote reductase (DHFR).^4^, ^5^ Pterin‐4α‐carbinolamine dehydratase (PCD) and DHPR enzymes are essential for BH4‐cofactor recycling.^2^, ^5^ Deficiency in any of these enzymes results in BH4 metabolism disorder.

Despite the differences in biochemical properties, similar clinical features are seen in variable severities at patients. Poor sucking, floppy baby, hypotonia of the trunk, hypertonia of the extremities, swallowing difficulties, myoclonic seizures, oculogyric crises, behavioral problems, intellectual disability, poor response to phenylalanine (Phe) limited diet can be seen. Hyperphenylalaninemia (HPA) occurs in the deficiency of autosomal recessive (ar) GTPCH (OMIM: 233910), PTPS (OMIM: 261640), DHPR (OMIM: 261630) and PCD (OMIM: 264070) whereas deficiency of SR (OMIM: 612716), autosomal dominant (ad) GTPCH (OMIM: 128230) and some ar GTPCH (OMIM: 233910) are presented without HPA. ^5^, ^6^

The primary objective of this retrospective study is to describe the demographics, diagnosis, clinical, biochemical, and molecular data of treatment and follow‐up of patients with BH4 deficiency. We believe that our study supports an expanding phenotypic spectrum and optimization of treatment in BH4 deficiency.

The patients' information was not included in any prior study.

## 2 PATIENTS AND METHODS

The presented retrospective single‐center study, included patients with BH4 deficiency, followed up from 2006 to 2019. Demographic, clinical features, laboratory and radiological findings, treatment and follow‐up information of nine patients with BH4 deficiency were enrolled. Anthropometric measurements were compared with healthy children of the same age and gender.^7^ First admitting Phe levels (measured by an high‐performance liquid chromatography [HPLC]) method (Immuchrom GmbH), where HPLC separation was performed by an isocratic method and UV dedector, blood pterin profile,^8^ DHPR activity in dried blood spots,^9^ plasma prolactin levels (measured by electrochemiluminescence immunoassay “ECLIA” [Roche Diagnostics GmbH]), and cerebrospinal fluid (CSF) pterins and biogenic amine analyses^8^, ^10^ were evaluated. Neurotransmitter metabolites 5‐hydroxindolacetic acid (5‐HIAA) and homovanillic acid (HVA) were measured to determine disease severity and monitor therapy.

BH4 loading test was performed in patients with blood Phe concentration >10 mg/dL at baseline; by measuring Phe levels at the 0, 4th, 8th, and 24th hours after ingestion of 20 mg/kg sapropterin.^4^ The patients' diagnoses were confirmed with genetic mutation analysis. Information related to the signs and symptoms of BH4 deficiency, treatment outcomes and adverse effects, magnetic resonance imaging (MRI) and electroencephalography (EEG) reports were collected. Intellectual assessment was assessed by Wechsler Intelligence Scale for Children‐Revised (WISC‐R), physical development status assessed using the Ankara Developmental Screening Inventory (ADSI).^11^

Genomic PCR and Sanger sequence analysis of genomic DNA isolated from blood samples was performed. We tested all exons of the corresponding genes, that is, *GCH1*, *PTS*, *SPR*, and *QDPR* plus their flanking intronic regions, with the following genomic reference sequences (cDNA in parenthesis) ENSG00000131979 (ENST00000491895.7), ENSG00000150787.3 (ENST00000280362.3), ENSG00000116096 (ENST00000280362.3), ENSG00000151552.6, respectively, and cDNA reference sequences, ENST00000280362.3, ENST00000234454.6, and ENST00000281243.5 respectively.

## 3 RESULTS

### 3.1 Patients

Nine patients (8/1—F/M) from eight families were included in the study. Seven (77%) individuals in our cohort were born to consanguineous parents. One (11%) with ar GTPCH deficiency, two (22%) with PTPS deficiency, three (34%) with SR deficiency, and other three (34%) patients were diagnosed with DHPR deficiency. The most frequent defects were SR and DHPR deficiencies. All three SR deficiency patients were offspring of a consanguineous first cousin marriage, that is, two siblings and their cousin coming from the same big family so they carried the same mutation. All our patients with PTPS and DHPR deficiency had severe phenotypes. None of them had a history of prematurity. Mean birth weight was 2804 g (SD ± 471). All patients had normal head circumference and height at birth. Three patients had low birth weight, one with PTPS deficiency, and two with DHPR deficiency.

Mean age at diagnosis was 23.50 months (n = 6 SD ± 41.82) for BH4 deficiency with HPA and median age at diagnosis was 11.5 years (min: 1 to max: 19.6) for SR deficiency. Mean age at first clinical symptoms for BH4 deficiency with HPA was 3.75 months (SD ±2.53) while median age for SR deficiency was 8 months (min: 6 to max: 9). Newborn screening (NBS) was performed in all patients except patient (P) 6. HPA was detected in six of eight patients by NBS and referred to our center. In all six patients with HPA, high initial Phe levels were confirmed by HPLC. The first admission Phe levels determined in our center were presented in Table 1. The three of six patients with HPA were diagnosed before the age of 6 months and treatment was started. Two patients (P3, P8) were diagnosed with routine blood pterin analysis at the first admission. Differential diagnosis of PKU was performed to P2 due to suspicious BH4 test. P7, P9, and P1 were diagnosed at the age of 18 months, 9 years, 7 months, respectively. The detailed clinical characteristic features of the patients were presented in Table 1.

**TABLE 1:** Clinical features of the patients

P	Diagnosis	M/F	Consanguinity	Family history	Birth weight (g)	Age at first symptoms (month)	Neurological symptoms	First admission Phe levels (mg/dL)	Age at diagnosis (month)	Age at last visit (month)	Antropometric measurements at last visit (SDS)			Symptoms after treatment
											Weight	Height	Head C.	
P1	ar GTPCH	M	Yes^a^	No	2750	3	Hypotonia, oculogyric spasm, developmental delay, convulsion	15.1	7	14	−2.41	−0.49	−2.35	Decreased Oculogyric spasms, head hold, unsupported seating
P2	PTPS	F	Yes	No	3150	2	Oculogyric spasm, hypotonia	11.3	2.5	5	−1.22	−1.68	−1.69	Decreased oculogyric spasms
P3	PTPS	F	Yes^a^	No	2390	3	Hypotonia	22.1	3	86	0.77	−0.75	0.08	Stereotypical hand movements, slight intellectual disability
P4	SPR	F	Yes^a^	Yes^b^	3300	6	Oculogyric spasm, severe hypotonia, hypersomnia	1.2	12	20	1.25	0.67	−0.06	Unsupported walking, slight speech delay
P5	SPR	F	Yes^a^	Yes^c^	3400	9	Dystonia, gait abnormality, developmental delay	NA	138	141	1.1	0.61	0.74	Improvement in handwriting, l‐dopa induced dyskinesia
P6	SPR	F	Yes^a^	Yes^b^	3150	8	Dystonia, convulsion, dysarthria, developmental delay	NA	236	240	1.4	0.27	1.54	Improvement in handwriting, dysarthria, l‐dopa induced dyskinesia
P7	DHPR	F	No	No	2700	6	Hypotonia, convulsion	2.9	18	70	0.44	−1.09	−1.04	Slight intellectual disability
P8	DHPR	F	No	No	2300	2.5	Developmental delay	9	2.5	59	−1.31	−2.4	−0.17	Slight intellectual disability, walking abnormality
P9	DHPR	F	Yes	No	2100	6	Developmental delay, convulsion	8	108	156	−6	−6.9	−6	Severe developmental delay, spasticity, gastrostomy tube feeding

^a^ First‐degree cousin marriage.

^b^ P4 and P6 are siblings.

^c^ P5 is the cousin of P4 and P6.

Abbreviations: GTPCH, GTP cyclohydrolase I; DHPR, dihydropteridine reductase; PTPS, 6‐pyruvoyl‐tetrahydropterin synthase.

**FIGURE 1:**
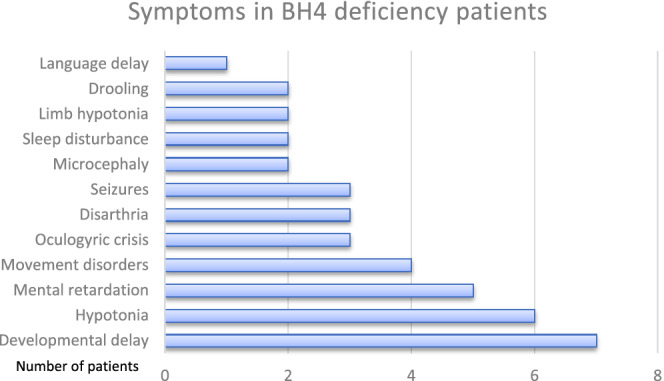
Clinical symptoms of the patients

P7 and P9 did not continue to follow‐up after first admission due to newborn screening. P7 was referred by general practitioner at the age of 18 months due to unable to walk. At last visit, she has slight intellectual disability. P9 was referred by a neurologist at the age of 9 when she developed neurological symptoms. She was diagnosed at 9 years old. She had global developmental delay, inability to stand, walk or even sit without assistance. At last visit she had axial hypotonia, hypertonia at the extremities and was fed via gastrostomy tube due to swallowing difficulties. She was on antiepileptic drugs. The patient had unilateral movement restriction at the right arm. P1 who was detected with HPA during newborn screening; however, he applied for the first time after the onset of neurological symptoms at the age of 7 months. He had severe hypotonia, seizures, oculogyric spasms, unable to hold head on admission. At last visit he had hypotonia, unsupported sitting, decreased oculogyric spasms. Hypersalivation, apathy, dysarthria, gait disturbance were exacerbated in P8 when blood Phe levels was high. Clinical symptoms worsened when the blood Phe levels was over 6 mg/dL. All three patients with SR deficiency were diagnosed after appearance of neurological symptoms. P4 had oculogyric spasms, generalised hypotonia, hypersomnia, unable to unsupported seating and hold her head at first admission. Clinical symptoms worsened in the evening before treatment. P5 and P6 had movement disorders. The most common clinical symptoms were developmental delay, intellectual disability, and movement disorders. Clinical symptoms were reported in Figure 1. At last visit, anthropometric measurements of seven (77.7%) of nine patients, including height, weight, and head circumference, were normal. Microcephaly was evident in P1 and P9 (22.2%).

### 3.2 Biochemistry

Mean blood Phe levels for BH4 deficiencies with HPA at the time of first admission was 11.4 mg/dL (SD: ±6.5, min: 2.9, max: 22.1). Before the start of the treatment, blood pterin levels and CSF analysis were performed. CSF 5‐HIAA and HVA levels were detected significantly low in CSF analysis. Molecular analysis were performed in all patients.

In ar GTPCH deficiency patient, low biopterin and neopterin levels were detected in both plasma and CSF. In PTPS deficiency high neopterin and low biopterin levels were detected in CSF analysis of one patient. In SR deficiency patients, biopterin and neopterin levels were normal in CSF and HVA and 5‐HIAA concentrations were significantly low in both two patients. In DHPR deficiency, all patients had normal pterin levels. DHPR activity was absent in two of three patients and very low in one. Due to the suspicious decrease in BH4 loading test, blood pterin were analyzed and revealed high neopterin, low biopterin, normal DHPR activity in P2 during the presymptomatic period. Neurological symptoms developed during the follow‐up. CSF could not be performed due to the lack of patient approval. All patients had high prolactin levels before treatment. Biochemical and molecular data are tabulated in Table 2.

**TABLE 2:** Biochemical and molecular data

Patient	Diagnosis	Blood neopterin	Blood biopterin	DHPR activity (N: 1.8‐3.8)	CSF neopterin (nmol/L)	CSF biopterin (nmol/L)	CSF HIAA	CSF HVA	CSF HVA/HIAA (1.5‐3.5)	CSF folic acid (ng/mL)	Initial blood prolactin (μg/L N:4.79‐23.3)	Molecular analysis
P1	ar GTPCH	0.06 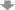 (N: 1.19‐2.93)	<0.01 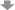 (N: 0.08‐1.20)	2.8 (N:1.8‐3.8)	3.48 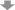 (N: 9‐40)	7.93 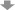 (N: 10‐50)	120 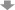 (N: 152‐462)	44 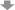 (N: 302‐845)	0.37 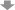	14.6	44.6	c.614 T > C (p.Val205Ala) HOMOZYGOUS
P2	PTPS	3.75 (N: 1.19‐2.93)	0.07 (N: 0.08‐1.20)	2.4 (N > 1,1)	NA	NA	NA	NA	NA	NA	29,83	c.84‐3C > G homozygous
P3	PTPS	1.8 (N: 0.35‐4.62)	0 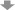 (N: 0.15‐1.66)	2.7 (N: 1.8‐3.8)	112 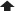 (N: 9‐40)	3.4 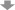 (N: 10‐50)	131 (N:100‐900)	252 (N:120‐500)	1.9	NA	85	c.200C > T (p.Thr67Met) homozygous
P4	SPR	NA	NA	NA	12.96 (N: 9–40)	41.69 (N: 10‐50)	16 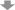 (N: 100‐900)	110 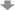 (N: 120‐500)	6.88 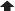	14.6	17.42	c.448A > G (p.Arg150Gly) homozygous
P5	SPR	0.21 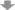 (N: 1.19–2.93)	0.10 (N: 0.08–1.20)	1.4 (N > 1.1)	28.12 (N: 9–40)	42.71 (N: 10‐50)	44.5 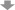 (N: 100‐900)	98 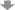 (N: 120‐500)	0.18 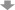	14	67	c.448A > G (p.Arg150Gly) homozygous
P6	SPR	NA	NA	NA	NA	NA	NA	NA	NA	NA	74	c.448A > G (p.Arg150Gly) homozygous
P7	DHPR	1.40 (N: 0.35–4.62)	1.49 (N: 0.15‐1.66)	0.1 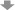 (N:1.8‐3.8)	13.1 (N:9‐30)	20.9 (N: 10‐30)	99.3 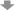 (N:105‐299)	<3 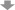 (N:211‐871)	–	NA	34.5	c.105C > G (p.Trp35Cys) homozygous
P8	DHPR	1.14 (N:0.35‐4.62)	0.93 (N: 0.15‐1.66)	0 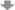 (N:1.8‐3.8)	NA	NA	NA	NA	NA	NA	37.56	c.291delC (p.Lys98Serfs*9) homozygous
P9	DHPR	1.2 (N: 0.35–4.62)	1.14 (N: 0.15‐1.66)	0 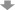 (N:1.8‐3.8)	NA	NA	NA	NA	NA	NA	34	c.661C > T (p.Arg221Ter) homozygous

Abbreviations: CSF, cerebrospinal fluid; DHPR, dihydropteridine reductase; GTPCH, GTP cyclohydrolase I; HIAA, hydroxindolacetic acid; HVA, homovanillic acid; PTPS, 6‐pyruvoyl‐tetrahydropterin synthase.

### 3.3 BH4 loading test

BH4 loading test, was performed in five patients. The patient with ar GTPCH deficiency (P6) had a 75% decrease in blood Phe levels 8 hours after oral BH4 administration. Two patients with PTPS deficiency had a 96.4% and 98.1% decrease in blood Phe levels, respectively, after BH4 administration during the same time period. Blood Phe levels were decreased by 51% and 55% in two patients with DHPR deficiency, respectively.

### 3.4 Central Nervous System Evaluation

Cranial MRI was performed in all patients. In patients with BH4 deficiency with HPA, the most common finding in cranial MRI was hyperintensity in T2‐Fluid attenuated inversion recovery sequences in the bilateral cerebral white matter. In addition, diffusion restriction was detected in P1. MRI was normal in all patients with SR deficiency. When EEGs of eight patients were evaluated, nonspecific EEG abnormality was detected in seven (87.5%) patients. Mild intellectual disability was detected in one patient whom were evaluated with WISC‐R and variable degrees of developmental delay were detected in four patients who were evaluated with ADSI.

### 3.5 Medical and diet treatment

All patients received l‐dopa (combination with benserazid) and 5‐hydroxytryptophan (5‐HTP). One patient with ar GTPCH and three patients with DHPR deficiency received Phe restricted diet. Diet therapy was not required in patients with PTPS deficiency. All BH4 deficiency patients with HPA, except P7 and P9 received sapropterin treatment. About 15 mg/d folinic acid were administered to the all patients with DHPR deficiency. Treatments and doses of patients are detailed in Table 3. Optimal therapeutic level was arranged according to the clinical response and plasma prolactin levels. One month after the start of treatment, P4 able to hold her head and unsupported seating. The significant decrease in the oculogyric spasm and hypotonia was noted. Melatonin was administered to the patient due to sleep disturbance for 1 month. Six months after the treatment, she was able to walk alone. At last visit she had mild speech delay. Although P5 and P6 with SR deficiency l‐dopa dose range was 2 to 4 mg/kg/d, dopa‐induced dyskinesia was observed. Since plasma prolactin levels were high, dopamine agonist (amantadine) was added to the treatment without decreasing the dose of l‐dopa. Dramatic improvement was observed in the dyskinesia of patients after amantadine. With l‐Dopa and 5‐HTP, vomiting occurred in one patient and diarrhea developed in one patient. Vomiting disappeared after 2 weeks without any intervention. Symptoms regressed with dietary changes in the patient with diarrhea.

**TABLE 3:** Treatment and dosages

Patient	Diagnosis	Phe restricted diet	l‐Dopa dose^a^ (mg/kg/day)	l‐Dopa recomended dose^b^ (mg/kg/day)	5‐HTP dose^c^ (mg/kg/day)	5‐HTP Recomended dose^a^ (mg/kg/day)	Bh4^d^ (mg/kg)	Folinic acid (mg/day)	Other	Side effects
P1	ar GTPCH	Yes	5	4‐7	4	3‐5	10.2		Baclofen levetiracetam	
P2	PTPS	No	6.9	4‐7	5.55	3‐5	15.4			
P3	PTPS	No	14.1	8‐15	9.4	6‐9	5.77	15		
P4	SPR	No	9.5	4‐10	9.6	3‐9				Vomiting
P5	SPR	No	4.5	4‐10	3.5	3‐9			Amantadine	Dopa‐induced dyskinesia
P6	SPR	No	4.46	4‐10	2.8	3‐9			Amantadine	Dopa‐induced dyskinesia
P7	DHPR	Yes	12.4	8‐15	8	6‐9		15		
P8	DHPR	Yes	9.77	8‐15	6.51	6‐9	5.6	15		
P9	DHPR	Yes	11.3	8‐15	7.1	6‐9		15	Levetiracetam	Diarrhoea

^a^ Previously published recommended doses (Ref. 4).

^b^ 1 mg/kg/day l‐dopa were initiated as four divided doses and dose increase of 1 to 2 mg/kg/day was made in 2 to 4 weeks intervals.

^c^ 1 mg/kg/day 5‐HTP was initiated as four divided doses and dose increase of 1 to 2 mg/kg/day was made in 2 to 4 weeks intervals.

^d^ Single dose of saptopterin,which varied, was given to patients.

Abbreviations: CSF, cerebrospinal fluid; DHPR, dihydropteridine reductase; GTPCH, GTP cyclohydrolase I; HIAA, hydroxindolacetic acid; HVA, homovanillic acid; PTPS, 6‐pyruvoyl‐tetrahydropterin synthase.

## 4 DISCUSSION

BH4 deficiencies are a heterogeneous group of pediatric neurometabolic disorders. The estimated incidence of BH4 deficiencies in the world is 1 to 2% of all patients with HPA (1:10 000).^12^, ^13^ The incidence of HPA is 1/4500 and BH4 deficiency is 2% of all patients with HPA in Turkey due to high rates of consanguineous marriages.^14^, ^15^ In our study, consanguinity was reported in six of eight (75%) families. Similarly, Coskun et al reported consanguinity rates as 85.7% (12/14) in BH4 deficiency patients with HPA in Turkey.^16^ There are geographical differences in the frequency of occurrence. Ye et al reported, among 256 cases of BH4 deficiency; 96% PTPS deficiency, 2.4% DHPR deficiency, and 1.6% AD GTPCH deficiency were observed.^17^ Opladen et al reported that, PTPS deficiency is the most frequent of all BH4 deficiencies (54%), followed by DHPR deficiency (33%).^10^ In contrast to international studies, the most common BH4 deficiency in Turkey is DHPR deficiency.^10^, ^16^, ^17^ Turgay et al reported that DHPR deficiency accounts for approximately 75% of the biopterin deficiencies in Turkey.^16^ In our study, the frequency of DHPR deficiency (34%) was the same as for SR deficiency (34%) (all patients were from the same family two siblings and their cousins).

Literature reports high incidence of prematurity and low birth weight in severe form of PTPS deficiency.^10^, ^18^ However, in our study, two of three low birth weight patients were DHPR and one was PTPS deficient leading to idea that severe form might have intrauterine effects.

As Opladen et al previously reported that the most common symptoms were developmental delay, abnormal muscle tone, and convulsions.^13^ The findings of our cohort (developmental delay, intellectual disability, and movement disorders) supported these data. Interestingly, in our cohort, microcephaly was detected in two patients, [ar GTPCH (P1), DHPR (P9) deficiencies] as it was reported by the literature.^6^, ^16^, ^17^ However, according to the literature, microcephaly is more common in PTPS and DHPR deficiency.

It is known that diagnosis in the neonatal period is crucial and the age of treatment initiation is determining the prognosis.^19^ In late diagnosed or untreated BH4 deficiencies, progressive neurological deterioration developed from the infantile period. Benign PCD deficiency is the exception of those. Coskun et al reported that the two cases showed a poor clinical course despite early diagnosis and treatment, and one of them died within 2 years.^16^ It is not yet known whether early treatment can completely prevent developmental delay in all patients with BH4 deficiency. In our study, developmental outcome is relatively poor in those treated after 6 months of age. Therefore, in the presence of unexplained neurological findings, cerebral palsy or developmental delay BH4 deficiency should be considered. To date prognosis is satisfying in the cases with early diagnose and treatment. Symptoms might have been exacerbated by increased levels of phenylalanine in BH4 deficiency patients with HPA. High phenylalanine levels affect the transport of neurotransmitter precursors through membranes and/or results in tyrosine and tryptophan hydroxylases competitive inhibition. Hypersalivation, apathy, dysarthria, and gait disturbance were exacerbated in DHPR deficiency when blood Phe levels was high. The patient's blood Phe levels are monitored at close intervals. Diurnal fluctuations can be seen in symptoms, seem to worsen in the evening.^13^, ^20^ Clinical symptoms of SR deficiency worsened in the evening before treatment.

Aiming to expand phenotype‐genotype correlation, we should share that; in ar GTPCH novel variant as a novel missense mutation c.614 T > C resulting in a V205G substitution has been identified in the *GCH1* gene. However, this patient had a low biopterin and neopterin levels in both plasma and CSF known as typical pattern. The homozygous mutations c.200C > T (p.Thr67Met) and c.84‐3C > G previously reported as severe form mutations from Albania, Italy, and Iran were detected in our PTPS deficiency patients who had normal neopterin and low biopterin levels in plasma.^21^, ^22^, ^23^, ^24^

In our cohort, all SR deficiency patients had normal CSF pterin but HVA and 5‐HIAA were significantly decreased. Homozygous c.448A > G (p.Arg150Gly) mutations previously reported as a common in Mediterranean region was detected in our patient. ^25^ No clear genotype‐phenotype correlation is apparent in SR deficiency.

DHPR deficiency, confirmed by c.105C > G (p.Trp35Cys), c.661C > T (p.Arg221Ter) and c.291delC (p.Lys98Serfs*9) homozygous mutations detected at *QDPR* gene. The c.105C > G (p.Trp35Cys) was not previously published. The mutation c.661C > T (p.Arg221Ter) was previously suggested to be common in patients of Mediterranean origin and also in Turkey.^26^, ^27^, ^28^ The c.291delC (p.Lys98Serfs*9) homozygous mutation identified in this study is predicted to cause premature termination of protein synthesis. Patient with c.661C > T (p.Arg221Ter) mutation has the most severe clinical features such as severe developmental delay, growth retardation, microcephaly, focal neurological deficit, swallowing difficulties, and epilepsy. However, due to the late diagnosis, it will not be appropriate to evaluate a genotype‐phenotype correlation.

Epilepsy is reported more frequently in DHPR deficiency than in other biopterin deficiencies.^2^, ^13^ Supporting these data, two from our three late diagnosed DHPR patients showed severe developmental delay. Although not usually associated with clinical impairment, abnormal EEGs can reveal the presence of CNS dysfunction, so EEG evaluation is an important assessment of these diseases. ^10^, ^29^

The most common finding in our patients MRI was hyperintense lesions on T2‐weighted images (5/9), correlated with reports from the literature about hyperintense lesions at the periventricular white matter.^23^, ^30^, ^31^, ^32^ Interestingly, in our cohort, calcifications in basal ganglion were not detected even in DHPR deficiency particularly accompanied with it.^29^, ^30^ In spite that Freidman et al reported that some of SR deficiency patients might have had hyperintense lesions before treatment, cranial MRI were generally normal in most patients with SR deficiency similarly to our MRI reports.^33^

All patients received l‐dopa and 5‐HTP. BH4 was used for the treatment in ar GTPCH and PTPS deficiencies. We administered BH4 treatment at four (66%) of six patients with HPA. DHPR deficiency has a special position because there were insufficient data to support the efficacy and safety of BH4 for DHPR deficiency patients due to the accumulation of 7,8‐dihydrobiopterin.^2^, ^4^ Repeated CSF analysis for HVA and 5‐HIAA is recommended for therapeutic monitorization; however, CSF sampling is invasive and often difficult to perform. In contrast, Jaggi et al reported that did not find correlation between CSF 5‐HIAA, HVA values and clinical outcome.^19^ Replacement therapy for dopamine is more difficult than adjusting the dose of serotonin due to the short half‐life and adverse effect. Monitoring serum morning prolactin levels as it was recommended combined with clinical findings was a successful way for optimising the l‐dopa dosage.^34^, ^35^, ^36^, ^37^ This may indicate the importance of monitoring CSF folates and folinic acid substitution in patients with PTPS deficiency. Folinic acid was started in one of our PTPS deficiency at the age of seven; due to movement disorders, despite high dose l‐dopa and 5‐HTP treatment. After the folinic acid was started, the patient's movement disorder relatively decreased.

The limitations of our retrospective study include loss of follow‐up, missing data and small number of cases.

The overall outcome in patients with BH4 deficiency is quite variable. BH4 metabolism disorder should be considered in the presence of a developmental delay, tonus abnormalities, intellectual disability, and movement disorders as it is also recommended in the guideline. Our experience in early treated cases suggests that clinical response may be satisfactory in BH4 deficiency. It is essential to evaluate both newborns presenting with hyperphenylalaninemia and patients with unknown neurological origin in terms of BH4 deficiencies.

## CONFLICT OF INTEREST

Ayse Ergul Bozaci, Esra Er, Havva Yazici, Ebru Canda, Sema Ucar Kalkan, Merve Saka Guvenc, Cenk Eraslan, Huseyin Onay, Sara Habif, Beat Thony, Mahmut Coker declare that they have no conflict of interest.

## AUTHOR CONTRIBUTION

Ayse Ergul Bozaci, Sema Kalkan Uçar, and Beat Thöny participated in the conception and design of the study, analysis and interpretation of data, drafting the manuscript, and in the critical revision of the manuscript for important intellectual content. Esra Er, Havva Yazici, Ebru Canda, and Mahmut Coker contributed to the analysis and interpretation of data and to the critical revision of the manuscript for important intellectual content. Merve Saka Guvenc, Cenk Eraslan, Huseyin Onay, and Sara Habif participated in the analysis and interpretation of data. All authors approved the submission.

## ETHICS STATEMENT

All procedures followed were in accordance with the ethical standards of the responsible committee on human experimentation (institutional and national) and with the Helsinki Declaration of 1975, as revised in 2000 (5). Informed consent was obtained from all patients for being included in the study. Additional informed consent was obtained from all patients for which identifying information is included in this article.
